# Effects of complex training on jump performance and neuromuscular activation in volleyball athletes: A controlled study

**DOI:** 10.1371/journal.pone.0347195

**Published:** 2026-04-10

**Authors:** Chen Chen, Ying Wang, Yu Hou, Pengfei Nie

**Affiliations:** 1 Department of Physical Education, Kunsan National University, Gunsan-si, Jeollabuk-do, South Korea; 2 Physical Education Department, Yanshan University, Qinhuangdao-si, Hebei, China; 3 School of Education, Woosuk University, Wanju-gun, Jeollabuk-do, Republic of Korea; 4 School of Fundamental Education, Nantong Institute of Technology, Nantong-si, Jiangsu, China; Università degli Studi di Milano: Universita degli Studi di Milano, ITALY

## Abstract

This study examined the integrated effects of complex training combining barbell back squats (BBS) and drop jumps (DJ) on jump performance and concentric-phase neuromuscular activation in volleyball athletes. In a randomized controlled design, twenty male volleyball athletes were allocated to an intervention group (IG, n = 10) or a control group (CG, n = 10). The IG completed an 8-week, twice-weekly complex training protocol (BBS followed by DJ), with loads progressively adjusted according to individual 1RM values, whereas the CG performed an intensity-matched BBS protocol. Primary outcomes were countermovement jump (CMJ) and approach jump (AJ) height and propulsion impulse (expressed in BW·s), while concentric-phase integrated electromyography (iEMG) and root mean square (RMS) amplitude were secondary outcomes. The results showed that, compared with the CG, the IG exhibited greater improvements in both CMJ and AJ height (CMJ: Δ = 6.13 vs 2.65 cm; AJ: Δ = 7.28 vs 2.78 cm; both *p* < 0.05). In addition, gains in propulsion impulse were larger in the IG (CMJ: Δ = 0.06 vs 0.02 BW·s; AJ: Δ = 0.08 vs 0.03 BW·s; both *p* < 0.05). Furthermore, electromyography revealed task-specific recruitment patterns: under BBS, the quadriceps and tibialis anterior showed higher iEMG and RMS values, whereas under DJ the lateral and medial heads of the gastrocnemius were more strongly activated, indicating complementary effects across key lower-limb muscle groups. Taken together, these findings indicate that an 8-week BBS–DJ complex program, compared with resistance training alone, is more effective in improving jump performance and propulsion impulse and in optimizing task-specific concentric activation patterns of the lower-limb musculature in volleyball athletes, and may serve as a feasible and evidence-based prescription for volleyball-specific conditioning.

## 1. Introduction

Volleyball is a net-based, opposition sport characterized by high-intensity jumping, brief bursts of power, and rapid transitions [1]. Net dominance at the front court, spiking, and blocking depend critically on vertical jump performance and the ability to execute repeated jumps across rallies [1,2]. Match analyses indicate that athletes often perform multiple jump types within a single rally, and fluctuations in jump capacity directly affect offensive–defensive efficiency and rally outcomes [1]. Mechanistically, jumping is not determined by force or velocity alone but emerges from coordinated multi-joint actions of the lower limbs within the stretch–shortening cycle (SSC), wherein eccentric absorption transitions to concentric output, modulated by the neuromuscular system’s temporal recruitment and control of motor units [3]. Consequently, prescriptions emphasizing either “strength” or “power” alone often fail to yield a stable transfer from strength to velocity to performance, underscoring the need for training strategies that jointly develop maximal strength and efficiently exploit the SSC [4].

In traditional practice, resistance training (e.g., barbell back squat [BBS]) enhances maximal strength and muscle cross-sectional area through high mechanical tension [5], while plyometric (jump) training (e.g., drop jump [DJ]) strengthens elastic recoil and neural drive via short ground contact and high stretch rates to augment the SSC [6]. Each modality has limitations. Resistance training transfers suboptimally to explosive velocity and contact-time reduction, whereas plyometrics are constrained by an insufficient strength base and technical proficiency. To integrate their advantages, complex training sequences high-intensity resistance exercise with explosive jumping within a single session or short window, using the former to “pre-activate” the neuromuscular system and thereby facilitate a more efficient transfer of strength to power [4,6]. This acute effect is commonly termed post-activation performance enhancement (PAPE), a transient improvement in voluntary performance following a conditioning activity. Proposed mechanisms include increased neural drive and changes in muscle contractile properties [7]. In the present study, the intervention is referred to consistently as complex training, whereas PAPE is used only to describe the acute performance-enhancing effect associated with a conditioning activity, rather than the training mode itself. Neurophysiological and biomechanical evidence indicates that high-intensity resistance exercise preferentially recruits high-threshold motor units, elevates neural drive, and increases musculotendinous stiffness [8]. Subsequent explosive tasks leverage the SSC for elastic energy storage and release and benefit from short-term gains via PAPE, thereby increasing power output per unit time, shortening contact time, and shifting the force–velocity relationship rightward [7,9,10]. Practically, the “BBS followed by repeated DJ” sequence is a canonical pairing with similar movement chains and clear temporal logic: BBS supplies high tension and extensor-chain strength reserves, whereas DJ enhances SSC efficiency and neuromotor rhythmic control under high stretch rates and brief contacts; their coupling within a session theoretically favors a continuous transfer from strength to velocity to performance [4,11].

Despite its widespread application, evidence on complex training remains limited in several respects. First, jump height and maximal strength are commonly used practitioner performance metrics in strength and conditioning [12,13]. However, many studies treat them as solitary endpoints when evaluating complex training [14,15]. With limited concurrent quantification of kinetics and electromyography, it is difficult to identify the mechanistic neuromechanical drivers of performance gains. Notably, propulsion impulse (normalized to body weight, BW·s), which integrates the force–time history during the propulsion phase and is theoretically closely related to jump height, is often overlooked [16^,^^17^]. Concentric-phase integrated electromyography (iEMG) and root mean square (RMS), which reflect recruitment and amplitude characteristics, are also infrequently examined in synchrony with kinetics [18,19]. Second, evidence on sport-specific tasks is insufficient, particularly for the approach jump (AJ), where adaptations related to coupling of horizontal momentum with vertical kinetics, contributions of the ankle plantar-flexor chain, and contact-time regulation are sparsely reported. By contrast, the countermovement jump (CMJ), although indicative of baseline explosiveness, still departs from the movement-chain demands of competition [2,20,21]. Third, heterogeneity in intervention duration, loading progression, exercise sequencing, and inter- and intra-set rest intervals compromises the comparability and generalizability of training prescriptions across studies [4,9]. Fourth, EMG investigations often focus on the quadriceps while underrepresenting the roles of the ankle plantar-flexor chain (lateral and medial heads of the gastrocnemius) and the dorsiflexor chain (tibialis anterior) in SSC efficiency and contact-time control, despite their critical importance for force transmission and energy conversion [16,21].

Addressing these gaps, we conducted a randomized controlled trial in trained volleyball athletes to compare an 8-week, twice-weekly complex training protocol (BBS followed by DJ) with an intensity-matched BBS-only program, examining effects on jump performance and neuromuscular characteristics. To capture both outcomes and mechanisms, we implemented a two-tier evaluation framework. (1) CMJ/AJ height and propulsion impulse (BW·s) as primary endpoints to index jump performance and explosive output [22]; (2) Concentric-phase iEMG and RMS as secondary endpoints to characterize task-specific recruitment of key muscles (vastus medialis, rectus femoris, vastus lateralis, biceps femoris, tibialis anterior, lateral and medial heads of the gastrocnemius).

Accordingly, we hypothesized that: (1) compared with the control group, the complex training group would achieve greater improvements in CMJ/AJ height and propulsion impulse; (2) EMG features would exhibit task-specific, complementary recruitment, BBS emphasizing the knee-extensor/stabilizer chains (e.g., quadriceps, tibialis anterior), whereas DJ emphasizing high-amplitude recruitment of the ankle plantar flexors chain (gastrocnemius heads); (3) these neuromuscular changes would align with kinetic gains—particularly in impulse—supporting the putative pathway from strength reserves to SSC efficiency and, ultimately, performance enhancement [4,11,16]. Overall, this study aims to provide integrated evidence along the chain from training prescription through neuromuscular adaptation and kinetic processes to performance outcomes, thereby advancing the scientific rigor and precision of volleyball-specific conditioning.

## 2. Materials and methods

### 2.1. Participants

Twenty volleyball athletes with a formal training background were recruited for this study between November 20, 2024, and January 20, 2025. No formal a priori power analysis was conducted before recruitment. The sample size was determined pragmatically based on athlete availability, eligibility criteria, and the controlled team-training context of the study. After being matched according to their physical condition and training experience, participants were randomly allocated in a 1:1 ratio to the intervention group (IG) or control group (CG) by lottery draw, with 10 participants in each group. All participants volunteered, provided written informed consent, and were capable of completing all study tasks [23]. Participant demographic and baseline physical characteristics are presented in Table 1. Prior to the intervention, independent-samples t tests were conducted for age, height, body mass, body mass index (BMI), training years, and BBS one-repetition maximum (1RM); results indicated no significant between-group differences (*p* > 0.05), supporting appropriate allocation and baseline comparability. Determination and use of the one-repetition maximum (1RM) followed prevailing standards in strength and conditioning [24]. The study was reviewed and approved by the Yanshan University Research Ethics Committee (Approval date: 2024−1120) and conducted in accordance with the Declaration of Helsinki.

**Table 1:** Means and standard deviations of anthropometric data for the intervention group (IG) and control group (CG).

Basic information	IG	CG	*P*
Age（years）	22.10 ± 2.69	22.20 ± 2.52	0.88
Height(m)	189.50 ± 6.04	186.50 ± 3.66	0.172
Body mass（kg）	82.50 ± 9.37	81.20 ± 9.54	0.746
Training Years（years）	7.4 ± 2.45	7.5 ± 2.54	1.000

### 2.2. Experimental design

The intervention lasted 8 weeks with two sessions per week (Tuesdays and Fridays). All sessions were fully supervised by coaches and investigators to ensure technical execution, protocol adherence, and training safety [23,24]. Attendance was recorded for all sessions to monitor compliance. Training loads were individualized based on baseline one-repetition maximum (1RM) testing and prescribed at 75%–85% 1RM, with the same relative progression scheme applied across participants. In the intervention group (IG), athletes performed a complex sequence of barbell back squat (BBS) followed by repeated drop jump (DJ), with 30 s between exercises and 3 min between sets. Repeated DJ was performed using a descending box-height sequence of 60, 45, 30, and 15 cm. The control group (CG) performed an intensity- and volume-matched BBS protocol with 3 min inter-set rest. A summary of the full training protocol is presented in Table 2.

**Table 2:** Structure of the intervention prescription by 2-week microcycles (per session).

Microcycle (Weeks)	IG: Main training (Load × Reps × Sets)	CG: Main training (Load × Reps × Sets)	Rest between sets	Interval (BBS → DJ)
Weeks 1–2	(75% 1RM BBS × 6 + repeated DJ) × 2 sets; (80% 1RM BBS × 5 + repeated DJ) × 5 sets; (85% 1RM BBS × 5 + repeated DJ) × 5 sets	(75% 1RM BBS × 6) × 2 sets; (80% 1RM BBS × 5) × 5 sets; (85% 1RM BBS × 5) × 5 sets	3 min	30 s (IG only)
Weeks 3–4	(75% 1RM BBS × 6 + repeated DJ) × 2 sets; (80% 1RM BBS × 5 + repeated DJ) × 5 sets; (85% 1RM BBS × 5 + repeated DJ) × 5 sets	(75% 1RM BBS × 6) × 2 sets; (80% 1RM BBS × 5) × 5 sets; (85% 1RM BBS × 5) × 5 sets	3 min	30 s (IG only)
Weeks 5–6	(75% 1RM BBS × 6 + repeated DJ) × 2 sets; (80% 1RM BBS × 5 + repeated DJ) × 5 sets; (85% 1RM BBS × 5 + repeated DJ) × 5 sets	(75% 1RM BBS × 6) × 2 sets; (80% 1RM BBS × 5) × 5 sets; (85% 1RM BBS × 5) × 5 sets	3 min	30 s (IG only)
Weeks 7–8	(75% 1RM BBS × 6 + repeated DJ) × 2 sets; (80% 1RM BBS × 5 + repeated DJ) × 5 sets; (85% 1RM BBS × 5 + repeated DJ) × 5 sets	(75% 1RM BBS × 6) × 2 sets; (80% 1RM BBS × 5) × 5 sets; (85% 1RM BBS × 5) × 5 sets	3 min	30 s (IG only)

BBS, Barbell Back Squat; DJ, Drop Jump; IG, intervention group; CG, control group. All sessions included a standardized warm-up (jogging + dynamic stretching + light BBS) and cool-down (foam rolling + static stretching). Repeated DJ was performed using a descending box-height sequence of 60, 45, 30, and 15 cm. All loads were prescribed as a percentage of the individual one-repetition maximum (1RM). The session structure was applied consistently throughout the 8-week intervention.

Grouping and prescription: IG performed a complex sequence of BBS followed by repeated DJ, with 30 s between exercises and 3 min between sets. The inter-exercise and inter-set rest intervals were selected with reference to the PAPE literature [9]. The CG performed an intensity- and volume-matched BBS protocol with 3 min inter-set rest. The CG protocol served as an active control representing conventional resistance training focused on developing maximal strength using a multi-joint exercise at moderate-to-heavy relative loads with adequate inter-set recovery, which is consistent with strength and conditioning guidelines for trained individuals. Accordingly, the between-group contrast primarily isolated the added effect of the plyometric component (repeated DJ) while matching the strength stimulus from BBS [25,26]. Session frequency, loading range, and number of sets were identical between groups; the only difference was the addition of repeated DJ in IG [4,9].

The standardized session structure was as follows: warm-up (jogging, dynamic stretching, and light BBS), main training sets (performed according to the loads and set schemes shown in Table 2), and cool-down (foam rolling and static stretching). In the IG, repeated DJ was performed immediately after BBS in a descending box-height sequence (60, 45, 30, and 15 cm), whereas the CG did not perform DJ [4,9].

BBS: stand naturally with feet slightly wider than shoulder width and toes externally rotated ~30°, maintaining an upright trunk and core stability. Descend until the thighs are parallel to the ground and pause for 1–2 s, then return to the start position via coordinated hip–knee–ankle extension, avoiding knee valgus and excessive trunk flexion; all repetitions were completed through a full range of motion (ROM) [24].

DJ: set four boxes (60 cm, 45 cm, 30 cm, 15 cm) with a center-to-center spacing of 60 cm. Jump with both feet, land forefoot first, rapidly absorb via hip and knee flexion and then perform a “quick extension” to re-jump, coordinated with arm swing and hip–knee–ankle extension, emphasizing minimal ground contact time (short contact and high stretch rate to reinforce the SSC) [27].

### 2.3. Data acquisition

The study consisted of two components: monitoring of the training process and evaluation of training effects. Mechanics during repeated DJ were collected in IG as process measures to identify key events for phase definition and EMG windowing/synchronization and to document execution characteristics of the conditioning activity; DJ mechanical variables were not treated as primary dependent outcomes for intervention effects. Training process monitoring focused on recording the neuromuscular (EMG) characteristics of the intervention group during the two training exercises, whereas the evaluation of training effects involved pre- and post-intervention testing in both the intervention and control groups. Based on the specific demands of volleyball, the key test movements selected were CMJ and one-step AJ [27,28].

#### 2.3.1. EMG acquisition.

EMG during propulsion was recorded as IG and CG performed BBS at 85% 1RM and, in IG, during repeated DJ. For BBS, the concentric phase spanned minimal knee flexion (bottom) to the end of ascent (upright stance) with both feet on the force plates. For CMJ, AJ, and DJ, propulsion spanned minimal knee flexion to take-off (vertical GRF = 0) [27].

#### 2.3.2. Countermovement jump testing.

Participants placed their hands on the hips (no arm swing) with each foot on a separate plate. On command, participants executed a maximal-effort CMJ and landed back on the plates with simultaneous foot contact and knee-flexion cushioning. Each participant performed three valid trials separated by 90s, and the maximum vertical center of mass (COM) height (cm, two decimals) was retained. Jump-height computation: using Visual3D, the COM height trajectory was obtained, and jump height was derived as the peak COM height minus the baseline COM height during quiet standing.

#### 2.3.3. Approach jump testing.

Participants stood on an approach platform with hands on the hips; the take-off foot contacted the force plates in sequence to complete take-off and landing, ensuring each foot contacted a separate plate for both take-off and landing. Each participant completed three valid trials separated by 90 s, and the maximum vertical jump height (cm, two decimals) was retained. Jump-height computation followed the same procedure as CMJ.

### 2.4. Data processing

#### 2.4.1. Kinematic data processing.

Kinematics were collected using a Qualisys A12 infrared high-speed three-dimensional motion capture system (8 cameras; sampling at 240 Hz). System calibration followed the Qualisys standard workflow, with residual error controlled within 0.8 mm. Based on the CAST marker set full-body model, forty-seven reflective markers (14 mm diameter) were placed on key anatomical landmarks of the trunk, pelvis, and limbs. Data were acquired in real time using Qualisys Track Manager (QTM; v2019.3, Qualisys AB, Gothenburg, Sweden) and exported to Visual3D for post-processing. Primary kinematic variables included knee joint angle, center of mass (COM) height, eccentric-phase duration, and propulsion-phase duration. Joint-angle data were filtered using a Butterworth low-pass filter (cut-off frequency of 10 Hz) to attenuate high-frequency noise [29].

#### 2.4.2 Surface EMG (sEMG) data processing.

A Delsys Trigno wireless electromyography system (Delsys Inc., Natick, MA, USA) was used at a sampling rate of 2000 Hz. Standard skin preparation was performed before acquisition, including shaving, alcohol cleaning/disinfection, application of conductive gel, and ensuring dryness. Electrodes were placed according to SENIAM guidelines on the principal lower-limb muscles: vastus lateralis (VL), rectus femoris (RF), vastus medialis (VM), biceps femoris (BF), tibialis anterior (TA), lateral head of the gastrocnemius (LG), and medial head of the gastrocnemius (MG); inter-electrode distance was 20 mm, with alignment along the muscle-fiber direction [30,31]. Raw EMG signals were denoised using a second-order Butterworth digital filter (10–450 Hz) and full-wave rectified. RMS was computed with a 50 ms sliding window, and iEMG was obtained by integrating the rectified signal within predefined phase windows. No MVIC-based normalization was performed; therefore, RMS and iEMG are reported in raw units (μV and μV·s, respectively) under standardized acquisition conditions.

Signal processing: raw EMG was denoised using a second-order Butterworth digital filter (10–450 Hz) and full-wave rectified, after which two time-domain metrics were extracted: (1) RMS, computed with a 50 ms sliding window (default overlap) to quantify intra-phase amplitude; and (2) iEMG, obtained by integrating the rectified signal within predefined phase windows (braking/propulsion) [18,31].

#### 2.4.3. Kinetic data processing.

Ground reaction force (GRF) during CMJ and AJ was synchronously acquired using four three-dimensional force plates (Kistler, Switzerland) at 2000 Hz. Zeroing of the Kistler force plates was performed prior to testing. Force signals were filtered with a second-order Butterworth low-pass filter (cut-off 50 Hz) to suppress high-frequency noise [29]. Propulsion impulse (I) was calculated as the product of vertical GRF (normalized to body weight) and propulsion-phase duration (I = F × t), and reported in BW·s[^28^]. Propulsion impulse was used as the primary kinetic metric to reflect the force–time integral during the propulsion phase, and EMG analysis windows were aligned to kinetic events to facilitate cross-modal interpretation [17,19]. All data streams were time-synchronized in QTM and Visual3D to ensure one-to-one correspondence across kinematics, kinetics, and EMG at key events. Synchronization was achieved using a TTL trigger via a synchronization hub to initiate and align recordings across the motion-capture system, force plates, and EMG system under a shared time base in QTM. Alignment was briefly verified by checking correspondence at identifiable events (e.g., take-off defined as vertical GRF = 0) across kinematics, kinetics, and EMG.

### 2.5. Statistical analysis

All statistical analyses were conducted using IBM SPSS Statistics 27.0.1 (IBM Corp., Armonk, NY, USA). Prior to inferential analyses, the Shapiro–Wilk test was used to assess normality for all variables. For between-group comparisons, homogeneity of variance was assessed using Levene’s test. Data are presented as mean ± standard deviation (SD). Within-group pre–post comparisons were performed using the paired-samples t-test. Between-group differences in intervention effects were examined using the independent-samples t-test. Because the main analyses were conducted using paired-samples and independent-samples t-tests rather than repeated-measures ANOVA, assumptions specific to repeated-measures ANOVA (e.g., sphericity) were not applicable. The significance level was set at p = 0.05 (two-tailed), with p < 0.05 considered statistically significant. Effect sizes were calculated as Cohen’s d for within-group pre–post changes (paired-samples) and for between-group differences in change scores (independent-samples). Effect size magnitudes were interpreted as small (0.2), moderate (0.5), and large (0.8). Ninety-five percent confidence intervals (95% CIs) were also reported for the principal effect estimates, including within-group mean differences and between-group differences in intervention effects. All figures were generated using OriginPro 2024b (OriginLab Corporation, Northampton, MA, USA).

## 3. Results

**Fig 1:**
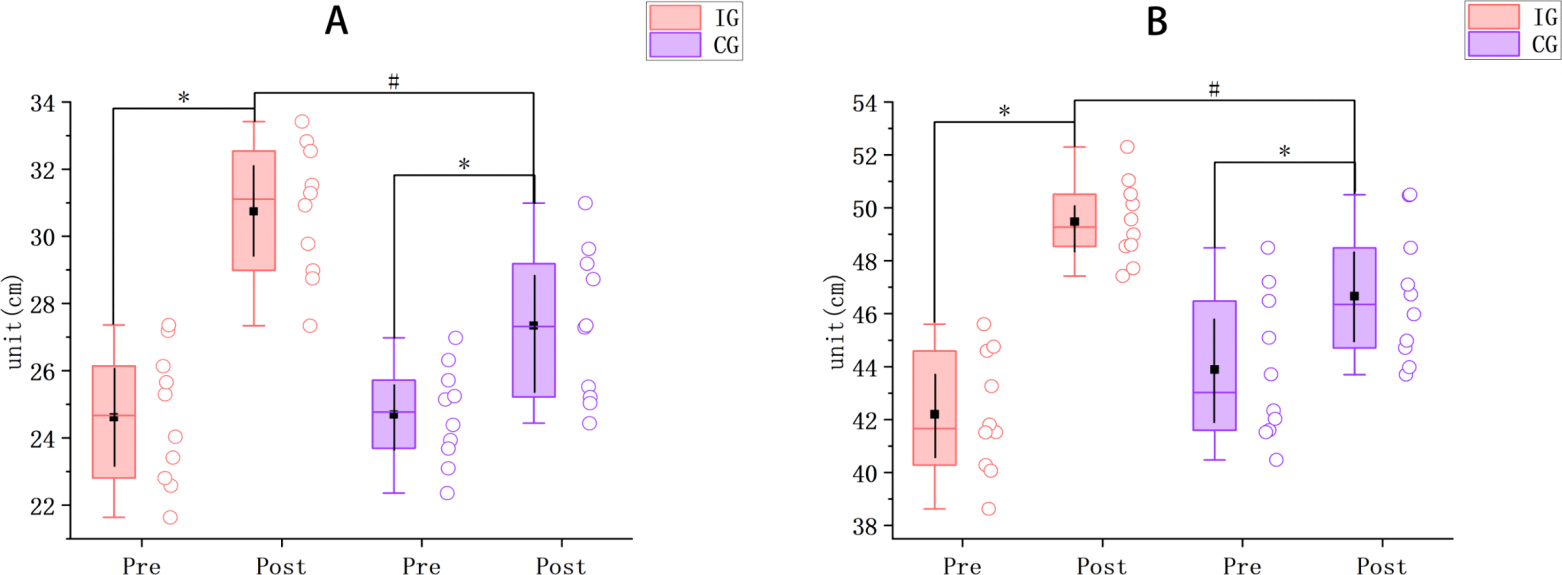
Changes in jump height. **(A)** Countermovement jump (CMJ); **(B)** Approach jump (AJ). Pre–post height changes are shown for the intervention group (IG) and the control group (CG) (units: cm). Open circles represent individual participant data, boxes indicate the interquartile range, center lines indicate the median, black squares indicate the mean, and vertical black lines indicate the 95% confidence interval. * indicates a significant within-group pre–post difference; # indicates a significant between-group difference in change (*p* < 0.05).

As shown in Fig 1, CMJ height in the IG increased from 24.61 ± 2.00 cm pre-intervention to 30.74 ± 1.98 cm post-intervention (Δ = 6.13 cm; 95% CI [5.32, 6.93]; p < 0.001; d = 5.46), whereas the CG increased from 24.69 ± 1.46 cm to 27.34 ± 2.25 cm (Δ = 2.65 cm; p < 0.001; 95% CI [1.89, 3.42]; d = 2.48). There were no significant between-group differences at baseline (*p* = 0.924; 95% CI [−1.72, 1.57]; d = −0.04); post-intervention, CMJ height was significantly greater in the IG than in the CG (*p* = 0.002; 95% CI [1.41, 5.39]; d = 1.61). In terms of magnitude, the improvement in the IG (6.13 cm) was greater than that in the CG (2.65 cm).

For AJ height, the IG increased from 42.20 ± 2.28 cm to 49.48 ± 1.53 cm (Δ = 7.28 cm; p < 0.001; 95% CI [6.58, 7.98]; d = 7.45), whereas the CG increased from 43.89 ± 2.76 cm to 46.67 ± 2.42 cm (Δ = 2.78 cm; p < 0.001; 95% CI [2.21, 3.33]; d = 3.52). No significant between-group differences were observed before the intervention (*p* = 0.153; 95% CI [−4.07, 0.69]; d = −0.67); post-intervention, AJ height was significantly higher in the IG than in the CG (*p* = 0.006; 95% CI [0.92, 4.72]; d = 1.39). Overall, the magnitude of improvement in the IG (7.28 cm) exceeded that in the CG (2.78 cm).

**Fig 2:**
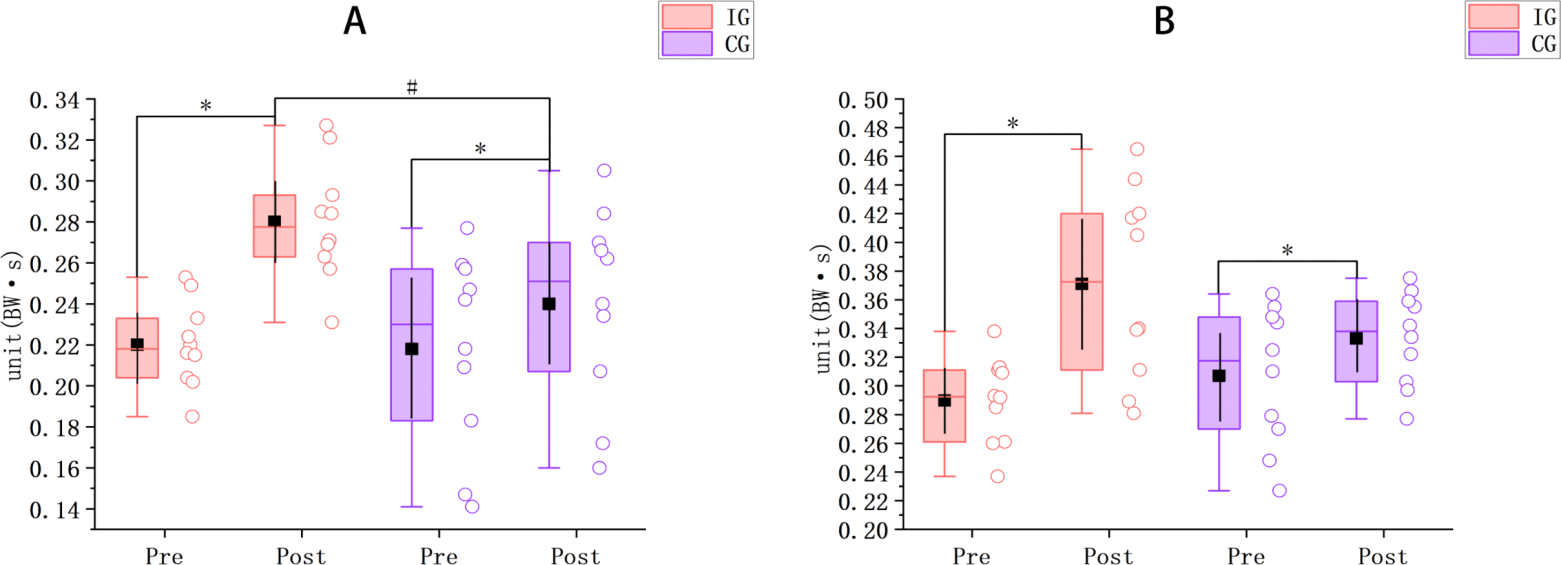
Changes in propulsion impulse. **(A)** CMJ; **(B)** AJ. Impulse is expressed in body-weight–normalized units (BW·s). Pre–post changes are presented for the IG and the CG. Open circles represent individual participant data, boxes indicate the interquartile range, center lines indicate the median, black squares indicate the mean, and vertical black lines indicate the 95% confidence interval. * indicates a significant within-group pre–post difference; # indicates a significant between-group difference in change *(p < 0.05)*.

As shown in Fig 2, for CMJ propulsion impulse, the IG increased from 0.22 ± 0.02 BW·s pre-intervention to 0.28 ± 0.03 BW·s post-intervention (Δ = 0.06 BW·s; *p* < 0.001; 95% CI [0.05, 0.07]; d = 5.58), whereas the CG increased from 0.22 ± 0.05 BW·s to 0.24 ± 0.05 BW·s (Δ = 0.02 BW·s; *p* < 0.001; 95% CI [0.02, 0.03]; d = 2.82). There were no significant between-group differences at baseline (*p* = 0.9; 95% CI [−0.03, 0.04]; d = 0.06); post-intervention, CMJ propulsion impulse was significantly greater in the IG than in the CG (*p* = 0.038; 95% CI [0.01, 0.07]; d = 1.00). In terms of magnitude, the improvement in the IG (0.06 BW·s) was greater than that in the CG (0.02 BW·s).

For AJ, the IG increased from 0.29 ± 0.03 BW·s to 0.37 ± 0.07 BW·s (Δ = 0.08 BW·s; *p* < 0.001; 95% CI [0.05, 0.11]; d = 2.21), whereas the CG increased from 0.30 ± 0.04 BW·s to 0.33 ± 0.03 BW·s (Δ = 0.03 BW·s; *p* < 0.001; 95% CI [0.02, 0.03]; d = 2.19). No significant between-group differences were detected at baseline (*p* = 0.324; 95% CI [−0.05, 0.02]; d = −0.45) or post-intervention (*p* = 0.12; 95% CI [0.01, 0.09]; d = 0.74); however, numerically, the improvement in the IG (0.08 BW·s) exceeded that in the CG (0.03 BW·s).

**Fig 3:**
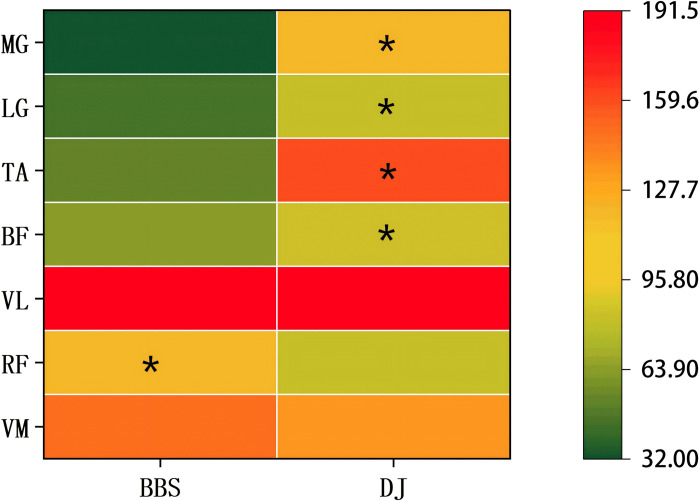
Concentric-phase root mean square (RMS) comparisons between barbell back squat (BBS) and drop jump (DJ) (units:μV). Muscles: Vastus medialis (VM), Rectus femoris (RF), Vastus lateralis (VL), Biceps femoris (BF), Tibialis anterior (TA), Lateral head of the gastrocnemius (LG), and Medial head of the gastrocnemius (MG). * indicates a significant difference between BBS and DJ (*p* < 0.05).

Comparing concentric-phase EMG amplitude between BBS and DJ (Fig 3), RF was significantly higher in BBS than in DJ (117.12 ± 26.00 vs 81.91 ± 22.00 μV; p < 0.001; 95% CI [31.48, 38.94]; d = 6.75). In contrast, BF, TA, LG, and MG were all significantly higher in DJ than in BBS (BF: 85.06 ± 22.00 vs 65.08 ± 20.00 μV, 95% CI [5.45, 34.50], d = 0.98; TA: 158.74 ± 15.00 vs 53.45 ± 10.00 μV, 95% CI [95.14, 115.44], d = 7.42; LG: 80.71 ± 14.00 vs 45.21 ± 12.00 μV, 95% CI [24.06, 46.93], d = 2.22; MG: 120.27 ± 16.00 vs 32.09 ± 8.00 μV, 95% CI [78.22, 98.14], d = 6.33; all *p* < 0.001). VM and VL showed no significant differences between conditions (VM: 150.35 ± 25.00 vs 135.01 ± 22.00 μV, *p* = 0.12; 95% CI [−5.06, 35.75]; d = 0.54; VL: 189.12 ± 30.00 vs 191.26 ± 32.00 Μv, *p* = 0.39; 95% CI [−3.31, 7.58]; d = 0.28). Overall, DJ elicited greater recruitment of the hamstrings and triceps surae, whereas BBS elicited stronger RF activation; VM and VL were comparable between tasks.

**Fig 4:**
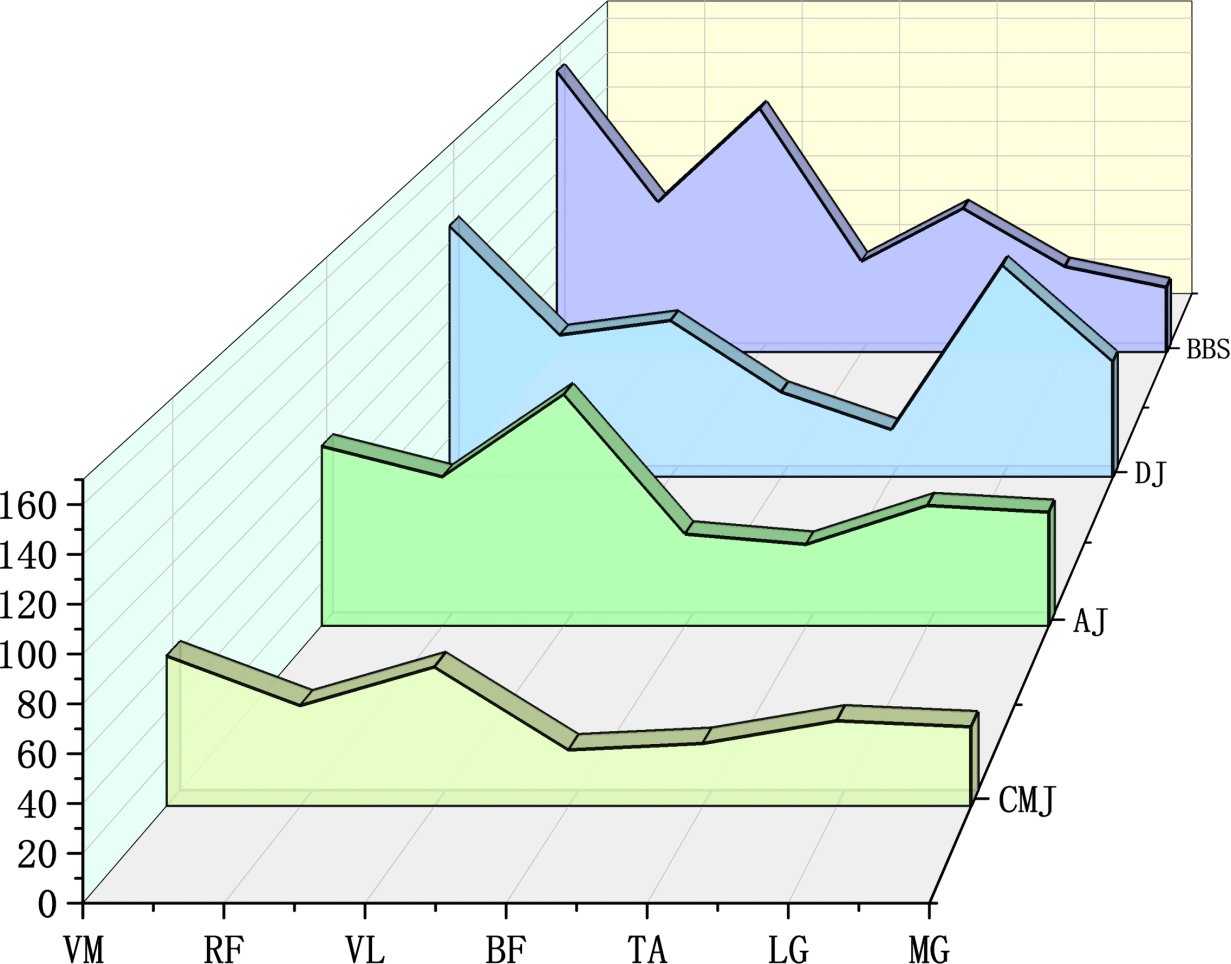
Concentric-phase integrated EMG (iEMG) comparisons across CMJ, AJ, BBS, and DJ (units:μV·s). Muscles as in Fig 3.

Effect sizes (Cohen’s d) are provided descriptively, and negative values indicate lower values in the first-listed condition. According to Fig 4, during BBS the iEMG values of VM (156.99 ± 22.00 μV·s), RF (83.88 ± 14.33 μV·s), VL (136.07 ± 18.00 μV·s), BF (50.99 ± 10.07 μV·s), and TA (80.43 ± 11.92 μV·s) were descriptively higher than during CMJ and AJ (VM: d = 4.25 vs CMJ and 3.09 vs AJ; RF: d = 2.12 and 1.15; VL: d = 4.13 and 1.01; BF: d = 2.97 and 1.31; TA: d = 4.86 and 3.11), indicating greater recruitment of the knee-extensor and dorsiflexor muscle groups under high-load resistance exercise. Conversely, the iEMG values of LG (48.03 ± 5.24 μV·s) and MG (36.47 ± 2.47 μV·s) were lower than during AJ (LG: d = −1.63; MG: d = −7.11). In contrast, DJ exhibited the opposite pattern: the iEMG values of LG (108.69 ± 12.00 μV·s) and MG (59.34 ± 9.50 μV·s) were higher than during CMJ and AJ (LG: d = 3.93 vs CMJ and 3.96 vs AJ; MG: d = 2.06 and 0.61), whereas those of VL, BF, and TA were lower than during BBS (VL: d = −12.45; BF: d = −2.17; TA: d = −13.27). These findings suggest that BBS preferentially reinforces recruitment of the knee-extensor and dorsiflexor groups, whereas DJ emphasizes recruitment of the gastrocnemius heads.

## 4. Discussion

Within an 8-week complex training protocol consisting of BBS combined with repeated DJ, we evaluated effects on volleyball athletes’ jump performance (CMJ and AJ), kinetic characteristics (propulsion impulse), and neuromuscular activation (iEMG and RMS). Compared with resistance training alone, the complex training group showed greater improvements in CMJ/AJ height and propulsion impulse, accompanied by task-specific changes in concentric-phase EMG activity, as reflected by iEMG and RMS [32,33].

Overall, the greater improvements in CMJ, AJ, and propulsion impulse observed in IG than in CG suggest that the classic association between the force–time integral (propulsion-phase net vertical impulse) and jump height was also evident in this cohort [34–36]. From a training perspective, BBS provides high mechanical tension and recruits high-threshold motor units, whereas the subsequent DJ emphasizes elastic recoil and rapid force production under short ground-contact times and high stretch rates within the SSC. When sequenced within the same training session, these characteristics may create conditions consistent with post-activation performance enhancement (PAPE), which has been associated with greater power output and shorter ground-contact time in previous studies [9,37]. Repeated exposure to this training configuration may therefore be related to favorable adaptations in the coordination of strength and velocity.

The EMG findings were generally consistent with this interpretation. iEMG indicated higher concentric-phase recruitment during BBS in the quadriceps (VM, RF, VL) as well as TA and BF, whereas DJ elicited stronger recruitment in LG and MG. These patterns suggest a complementary division of labor between the knee-extensor and ankle plantar flexor chains across tasks, aligning with evidence for phase-specific recruitment determined by distinct movement chains [33,35]. This task dependence is also consistent with evidence from female volleyball players showing that increases in drop-jump height did not necessarily produce significant differences in jump or neuromuscular markers, indicating that lower-limb EMG responses may vary according to protocol design and athlete characteristics [38]^.^ RMS results showed greater VM and RF amplitudes during BBS than during DJ, whereas DJ produced significantly higher RMS in LG/MG and TA. Together, these findings suggest that BBS places greater emphasis on the knee extensors and postural stabilization, whereas DJ is more closely associated with the contribution of the ankle plantar flexors to elastic energy utilization and rapid force transmission within the SSC. Increased RMS in this context may indicate altered neuromuscular recruitment characteristics associated with explosive performance; however, variables such as motor-unit synchrony and neural drive efficiency were not directly measured in the present study and therefore should be interpreted with caution [39].

Furthermore, the greater improvement in AJ relative to CMJ suggests that, under dynamic conditions requiring coupling of horizontal momentum with vertical kinetics, complex training may have a stronger integrative association with multi-joint torque coordination and ground-reaction-force timing. Prior studies likewise indicate that, in tasks such as vertical and approach jumps, optimization of the force–velocity profile and mechanical output characteristics accompanies performance enhancement [36,40]. In addition, the short ground-contact time and rapid transition characteristics of DJ may be conducive to more efficient SSC utilization and a greater contribution of the ankle plantar flexors. In this context, the ankle joint plays a pivotal role in jump tasks [35,41].

These findings are broadly consistent with meta-analytic evidence showing that complex and contrast training can improve jump-related performance in team-sport athletes, although the magnitude of benefit is not uniformly superior to more traditional training models [15,42,43]. However, the literature is not fully consistent in volleyball-specific contexts. Berriel et al. reported that, in elite male volleyball players, the addition of a heavy resistance stimulus to jump training did not produce greater improvements in vertical jump performance than jump training alone [44]. Likewise, Masel and Maciejczyk observed no acute PAPE effect in elite male volleyball players under a complex training protocol, suggesting that potentiation responses may depend on athlete level, protocol design, and recovery organization [45]. Taken together, these findings indicate that the effectiveness of complex training and PAPE-based strategies is likely context-dependent rather than uniform across all volleyball populations and training settings.

At the neurophysiological level, prior literature has proposed that PAPE may be associated with mechanisms such as augmented Ca² ⁺ release from the sarcoplasmic reticulum following high-intensity resistance exercise and increased myosin light-chain phosphorylation. These processes may transiently enhance subsequent force production and shortening velocity. Repeated exposure to such stimuli has also been discussed as a potential contributor to longer-term neuromuscular adaptation [7,37]. Nevertheless, these upstream mechanisms were not directly assessed in the present study. Accordingly, they should be regarded as theoretical explanations derived from previous literature rather than mechanisms directly verified by the current data. In the present context, the observed improvements in jump height, propulsion-related indicators, and EMG variables are more appropriately interpreted as being associated with task-specific neuromuscular adaptations under complex training.

From the perspective of movement-chain synergy, the present findings suggest that complex training may be associated with complementary task-specific adaptations across the anterior and posterior lower-limb chains. BBS primarily reinforces the knee-extensor and associated stabilizer chains, whereas DJ accentuates the ankle plantar flexor chain and the elastic response from landing to take-off. In combination, these features enhance energy-transfer efficiency and performance stability [32,33,40]. Thus, the present results should not be interpreted as direct proof of a single causal mechanism. Instead, they support the view that complex training is associated with coordinated changes in muscle activation patterns, movement-chain function, and jump-related performance.

From a practical standpoint, complex training may provide a useful option for periodized conditioning. Pairing high-intensity BBS and DJ in a fixed within-session sequence, with appropriate inter-exercise intervals to exploit the PAPE window, may help improve repeated rapid take-off performance and reactive actions relevant to volleyball competition. In particular, during spiking and blocking, repeated DJ exposure may be associated with improved neuromuscular readiness and more efficient ground-contact responses. Moreover, the concurrent use of iEMG and RMS in the present study offered complementary information for characterizing the neuromuscular responses associated with complex training, although these measures should be interpreted as indirect indicators rather than direct evidence of specific causal neurophysiological mechanisms.

This study has several limitations. First, the sample size was relatively small (n = 20), and no formal a priori power analysis was conducted before recruitment, which may limit statistical power and the generalizability of the findings. Second, only male volleyball athletes were included; therefore, the present results may not be directly generalizable to female players, who may differ in neuromuscular characteristics and training responses. Third, minor electrode displacement and changes in skin impedance across tasks may have affected EMG amplitude, and inter-individual variability in signal intensity may have influenced between-condition comparisons. In addition, EMG amplitudes were not normalized to MVIC, which may limit the comparability of absolute amplitude values across participants. Fourth, the 8-week intervention period and the absence of long-term follow-up do not allow conclusions regarding the durability of these adaptations over time. Finally, although the training protocol was implemented in athletes, it was conducted under a supervised and controlled study context, which may limit ecological validity relative to routine team training and competitive settings. Future research should include larger and more diverse samples, incorporate female athletes, extend follow-up periods, and integrate more upstream indicators, such as nerve conduction velocity, muscle-fiber type characteristics, and central neural drive, to clarify the mechanisms underlying complex training within a broader multi-level framework.

## 5. Conclusions

In this randomized controlled trial, an 8-week complex training protocol combining barbell back squat (BBS) with repeated drop jump (DJ) significantly improved lower-limb explosive performance and task-specific neuromuscular activation in volleyball athletes. The observed benefits of complex training may be partly related to acute responses consistent with PAPE, acting together with more efficient use of the stretch–shortening cycle (SSC). These adaptations may contribute to a more efficient transfer from strength to velocity and improved task-specific neuromuscular coordination. Compared with traditional resistance training, the complex training protocol produced greater gains in functional lower-limb performance and volleyball-specific jump outcomes, providing a practical and evidence-based option for volleyball-specific conditioning programs. Strength outcomes were not directly assessed in this study and therefore were not inferred.

## Supporting information

S1 Fig Experimental setup for barbell back squat (BBS) data collection. The photograph shows the equipment arrangement used during BBS testing (squat rack and barbell, force plate, motion-capture system, and EMG/synchronization hardware). Faces were anonymized.
(TIF)

S1 Data (XLSX)

## References

[1] Lima RF, Palao JM, Clemente FM. Jump Performance During Official Matches in Elite Volleyball Players: A Pilot Study. J Hum Kinet. 2019;67:259–69. doi: 10.2478/hukin-2018-0080 31523323 PMC6714353

[2] Vaverka F, Jandačka D, Zahradník D, Uchytil J, Farana R, Supej M, et al. Effect of an Arm Swing on Countermovement Vertical Jump Performance in Elite Volleyball Players: FINAL. J Hum Kinet. 2016;53:41–50. doi: 10.1515/hukin-2016-0009 28149409 PMC5260575

[3] Seiberl W, Hahn D, Power GA, Fletcher JR, Siebert T. Editorial: The Stretch-Shortening Cycle of Active Muscle and Muscle-Tendon Complex: What, Why and How It Increases Muscle Performance? Front Physiol. 2021;12:693141. doi: 10.3389/fphys.2021.693141 34093246 PMC8173190

[4] Thapa RK, Weldon A, Freitas TT, Boullosa D, Afonso J, Granacher U, et al. What do we Know about Complex-Contrast Training? A Systematic Scoping Review. Sports Med Open. 2024;10(1):104. doi: 10.1186/s40798-024-00771-z 39333341 PMC11436572

[5] Straub RK, Powers CM. A Biomechanical Review of the Squat Exercise: Implications for Clinical Practice. Int J Sports Phys Ther. 2024;19(4):490–501. doi: 10.26603/001c.94600 38576836 PMC10987311

[6] Ebben WP. Complex training: a brief review. J Sports Sci Med. 2002;1(2):42–6. 24688269 PMC3963241

[7] Blazevich AJ, Babault N. Post-activation Potentiation Versus Post-activation Performance Enhancement in Humans: Historical Perspective, Underlying Mechanisms, and Current Issues. Front Physiol. 2019;10:1359. doi: 10.3389/fphys.2019.01359 31736781 PMC6838751

[8] Aagaard P, Simonsen EB, Andersen JL, Magnusson P, Dyhre-Poulsen P. Increased rate of force development and neural drive of human skeletal muscle following resistance training. J Appl Physiol (1985). 2002;93(4):1318–26. doi: 10.1152/japplphysiol.00283.2002 12235031

[9] Xu K, Blazevich AJ, Boullosa D, Ramirez-Campillo R, Yin M, Zhong Y, et al. Optimizing Post-activation Performance Enhancement in Athletic Tasks: A Systematic Review with Meta-analysis for Prescription Variables and Research Methods. Sports Med. 2025;55(4):977–1008. doi: 10.1007/s40279-024-02170-6 39853660

[10] Seitz LB, Haff GG. Factors Modulating Post-Activation Potentiation of Jump, Sprint, Throw, and Upper-Body Ballistic Performances: A Systematic Review with Meta-Analysis. Sports Med. 2016;46(2):231–40. doi: 10.1007/s40279-015-0415-7 26508319

[11] Markovic G, Mikulic P. Neuro-musculoskeletal and performance adaptations to lower-extremity plyometric training. Sports Med. 2010;40(10):859–95. doi: 10.2165/11318370-000000000-00000 20836583

[12] Alba-Jiménez C, Moreno-Doutres D, Peña J. Trends Assessing Neuromuscular Fatigue in Team Sports: A Narrative Review. Sports (Basel). 2022;10(3):33. doi: 10.3390/sports10030033 35324642 PMC8950744

[13] Seo D-I, Kim E, Fahs CA, Rossow L, Young K, Ferguson SL, et al. Reliability of the one-repetition maximum test based on muscle group and gender. J Sports Sci Med. 2012;11(2):221–5. 24149193 PMC3737872

[14] Bauer P, Uebellacker F, Mitter B, Aigner AJ, Hasenoehrl T, Ristl R, et al. Combining higher-load and lower-load resistance training exercises: A systematic review and meta-analysis of findings from complex training studies. J Sci Med Sport. 2019;22(7):838–51. doi: 10.1016/j.jsams.2019.01.006 30683485

[15] Pagaduan J, Pojskic H. A Meta-Analysis on the Effect of Complex Training on Vertical Jump Performance. J Hum Kinet. 2020;71:255–65. doi: 10.2478/hukin-2019-0087 32148589 PMC7052715

[16] Biel P, Ewertowska P, Stastny P, Krzysztofik M. Effects of Complex Training on Jumping and Change of Direction Performance, and Post-Activation Performance Enhancement Response in Basketball Players. Sports (Basel). 2023;11(9):181. doi: 10.3390/sports11090181 37755858 PMC10534482

[17] Kirby TJ, McBride JM, Haines TL, Dayne AM. Relative net vertical impulse determines jumping performance. J Appl Biomech. 2011;27(3):207–14. doi: 10.1123/jab.27.3.207 21844609

[18] Arabadzhiev TI, Dimitrov VG, Dimitrova NA, Dimitrov GV. Interpretation of EMG integral or RMS and estimates of “neuromuscular efficiency” can be misleading in fatiguing contraction. J Electromyogr Kinesiol. 2010;20(2):223–32. doi: 10.1016/j.jelekin.2009.01.008 19233687

[19] Carvalho CR, Fernández JM, Del-Ama AJ, Oliveira Barroso F, Moreno JC. Review of electromyography onset detection methods for real-time control of robotic exoskeletons. J Neuroeng Rehabil. 2023;20(1):141. doi: 10.1186/s12984-023-01268-8 37872633 PMC10594734

[20] Tai WH, Peng HT, Song CY, Lin JZ, Yu HB, Wang LI. Dynamic characteristics of approach spike jump tasks in male volleyball players. Applied Sciences. 2021;11:2710. doi: 10.3390/app11062710

[21] Fuchs PX, Menzel H-JK, Guidotti F, Bell J, von Duvillard SP, Wagner H. Spike jump biomechanics in male versus female elite volleyball players. J Sports Sci. 2019;37(21):2411–9. doi: 10.1080/02640414.2019.1639437 31280702

[22] Eythorsdottir I, Gløersen Ø, Rice H, Werkhausen A, Ettema G, Mentzoni F, et al. The Battle of the Equations: A Systematic Review of Jump Height Calculations Using Force Platforms. Sports Med. 2024;54(11):2771–91. doi: 10.1007/s40279-024-02098-x 39425876 PMC11561012

[23] World Medical Association. World Medical Association Declaration of Helsinki: ethical principles for medical research involving human subjects. JAMA. 2013;310:2191–4. doi: 10.1001/jama.2013.281053 24141714

[24] Warneke K, Wagner C-M, Keiner M, Hillebrecht M, Schiemann S, Behm DG, et al. Maximal strength measurement: A critical evaluation of common methods-a narrative review. Front Sports Act Living. 2023;5:1105201. doi: 10.3389/fspor.2023.1105201 36873661 PMC9981657

[25] American College of Sports Medicine. Progression models in resistance training for healthy adults. Med Sci Sports Exerc. 2009;41(3):687–708. doi: 10.1249/MSS.0b013e3181915670 19204579

[26] Kraemer WJ, Ratamess NA. Fundamentals of resistance training: progression and exercise prescription. Med Sci Sports Exerc. 2004;36(4):674–88. doi: 10.1249/01.mss.0000121945.36635.61 15064596

[27] Anicic Z, Janicijevic D, Knezevic OM, Garcia-Ramos A, Petrovic MR, Cabarkapa D. Assessment of countermovement jump: what should we report? Life. 2023;13(1):190. doi: 10.3390/life13010190 36676138 PMC9865236

[28] Petrigna L, Karsten B, Marcolin G, Paoli A, D’Antona G, Palma A, et al. A Review of Countermovement and Squat Jump Testing Methods in the Context of Public Health Examination in Adolescence: Reliability and Feasibility of Current Testing Procedures. Front Physiol. 2019;10:1384. doi: 10.3389/fphys.2019.01384 31787902 PMC6853898

[29] Crenna F, Rossi GB, Berardengo M. Filtering Biomechanical Signals in Movement Analysis. Sensors (Basel). 2021;21(13):4580. doi: 10.3390/s21134580 34283131 PMC8271607

[30] Shapiro SS, Wilk MB. An Analysis of Variance Test for Normality (Complete Samples). Biometrika. 1965;52(3/4):591. doi: 10.2307/2333709

[31] Delsys I. Trigno wireless biofeedback system user’s guide. https://www.delsys.com/downloads/USERSGUIDE/trigno/wireless-biofeedback-system.pdf. 2021. Accessed 2025 November 19.

[32] Zhou L, Tan Y, Gan J, Li C, Bao D, Zhou J. Complex training with blood flow restriction increases power output and bar velocity during half-squat jump: a pilot randomized controlled study. Front Physiol. 2024;15:1368917. doi: 10.3389/fphys.2024.1368917 38883184 PMC11177751

[33] Rong B, Xiu C. Effects of Cluster vs. Traditional Sets Complex Training on Physical Performance Adaptations of Trained Male Volleyball Players. J Sports Sci Med. 2024;23(4):822–33. doi: 10.52082/jssm.2024.822 39649557 PMC11622053

[34] McBride JM, Kirby TJ, Haines TL, Skinner J. Relationship between relative net vertical impulse and jump height in jump squats performed to various squat depths and with various loads. Int J Sports Physiol Perform. 2010;5(4):484–96. doi: 10.1123/ijspp.5.4.484 21266733

[35] Panoutsakopoulos V, Bassa E. Countermovement Jump Performance Is Related to Ankle Flexibility and Knee Extensors Torque in Female Adolescent Volleyball Athletes. J Funct Morphol Kinesiol. 2023;8(2):76. doi: 10.3390/jfmk8020076 37367240 PMC10299299

[36] Molla RY, Fatahi A, Khezri D, Ceylan HI, Nobari H. Relationship between impulse and kinetic variables during jumping and landing in volleyball players. BMC Musculoskelet Disord. 2023;24(1):619. doi: 10.1186/s12891-023-06757-4 37516876 PMC10386276

[37] Olsen OR, Bojsen-Møller J, Aagaard P. Post-Activation Performance Enhancement in Strength and Power Sports-A Narrative Review. Scand J Med Sci Sports. 2025;35(11):e70162. doi: 10.1111/sms.70162 41195963

[38] Torres-Banduc M, Ramirez-Campillo R, Andrade DC, Calleja-González J, Nikolaidis PT, McMahon JJ, et al. Kinematic and Neuromuscular Measures of Intensity During Drop Jumps in Female Volleyball Players. Front Psychol. 2021;12:724070. doi: 10.3389/fpsyg.2021.724070 34616338 PMC8488207

[39] Besomi M, Hodges PW, Clancy EA, Van Dieën J, Hug F, Lowery M, et al. Consensus for experimental design in electromyography (CEDE) project: Amplitude normalization matrix. J Electromyogr Kinesiol. 2020;53:102438. doi: 10.1016/j.jelekin.2020.102438 32569878

[40] Jiménez-Reyes P, Samozino P, García-Ramos A, Cuadrado-Peñafiel V, Brughelli M, Morin J-B. Relationship between vertical and horizontal force-velocity-power profiles in various sports and levels of practice. PeerJ. 2018;6:e5937. doi: 10.7717/peerj.5937 30479900 PMC6238764

[41] Xu D, Zhou H, Wang M, Ma X, Gusztav F, Chon T-E, et al. Contribution of ankle motion pattern during landing to reduce the knee-related injury risk. Comput Biol Med. 2024;180:108965. doi: 10.1016/j.compbiomed.2024.108965 39084051

[42] Cormier P, Freitas TT, Rubio-Arias JÁ, Alcaraz PE. Complex and Contrast Training: Does Strength and Power Training Sequence Affect Performance-Based Adaptations in Team Sports? A Systematic Review and Meta-analysis. J Strength Cond Res. 2020;34(5):1461–79. doi: 10.1519/JSC.0000000000003493 32084104

[43] Lesinski M, Muehlbauer T, Büsch D, Granacher U. Effects of complex training on strength and speed performance in athletes: A systematic review. Sportverletz Sportschaden. 2014;28(2):85–107. doi: 10.1055/s-0034-1366145 24599505

[44] Berriel GP, Cardoso AS, Costa RR, Rosa RG, Oliveira HB, Kruel LFM, et al. Does Complex Training Enhance Vertical Jump Performance and Muscle Power in Elite Male Volleyball Players?. Int J Sports Physiol Perform. 2022;17(4):586–93. doi: 10.1123/ijspp.2021-0187 35130507

[45] Masel S, Maciejczyk M. No effects of post-activation performance enhancement in elite male volleyball players under complex training. Sci Rep. 2024;14(1):13708. doi: 10.1038/s41598-024-64604-5 38877057 PMC11178877

